# Subjective well-being in individuals with obsessive compulsive disorder: an exploratory study

**DOI:** 10.1192/j.eurpsy.2025.702

**Published:** 2025-08-26

**Authors:** R. B. Radins, J. V. B. Ferrão, J. P. B. Tomé, C. T. Reppold, Y. A. Ferrão

**Affiliations:** 1 PPGCS; 2 Psychiatry, UFCSPA; 3 Medicine, PUCRS; 4 Autism Treatment Reference Center, Porto Alegre City Hall, Porto Alegre, Brazil

## Abstract

**Introduction:**

Subjective well-being (SWB), defined as the way people think and feel about their lives, is often used to evaluate happiness. Separated into three components—positive affect, negative affect, and life satisfaction—SWB has been indirectly related to psychiatric symptoms and disorders. However, the relationship between SWB and obsessive-compulsive disorder (OCD) remains relatively unknown.

**Objectives:**

To determine whether SWB components correlate with the clinical features of OCD.

**Methods:**

This was a cross-sectional study evaluating 68 individuals with OCD. Sociodemographic data were collected, treatment histories were taken, and validated instruments were applied (Y-BOCS, Dimensional Y-BOCS, USP-Sensory Phenomena Scale, BDI-II, BAI, Positive and Negative Affect Schedule (PANAS), and Satisfaction with Life Scale (SWLS)).

**Results:**

All three SWB components showed inverse correlations with the severity of depressive/anxiety symptoms and total OCD symptom scores. Life satisfaction and positive affect showed inverse correlations with suicidality. Life satisfaction and negative affect showed inverse correlations with the hoarding and contamination/cleaning OCD dimensions, respectively. No SWB aspect correlated with any other OCD dimension.

**Conclusions:**

Some OCD-specific factors appear to correlate with SWB. However, it seems that the effect size is greatest for depressive symptoms, which suggests that depression mediates the relationship between OCD and SWB. Studies with larger samples and control groups are needed to confirm our findings.

**Disclosure of Interest:**

None Declared

